# Radiotherapy for geriatric head-and-neck cancer patients: what is the value of standard treatment in the elderly?

**DOI:** 10.1186/s13014-020-1481-z

**Published:** 2020-02-04

**Authors:** Erik Haehl, Alexander Rühle, Hélène David, Tobias Kalckreuth, Tanja Sprave, Raluca Stoian, Christoph Becker, Andreas Knopf, Anca-Ligia Grosu, Nils H. Nicolay

**Affiliations:** 1 Department of Radiation Oncology, University of Freiburg - Medical Center, Robert-Koch-Str. 3, 79106 Freiburg, Germany; 2 German Cancer Consortium (DKTK) Partner Site Freiburg, German Cancer Research Center (dkfz), Neuenheimer Feld 280, 69120 Heidelberg, Germany; 3 Department of Molecular and Radiation Oncology, German Cancer Research Center (dkfz), Neuenheimer Feld 280, 69120 Heidelberg, Germany; 4 Department of Otorhinolaryngology, University of Freiburg - Medical Center, Killianstr. 5, 79106 Freiburg, Germany

**Keywords:** Head-and-neck cancer, Head-and-neck squamous cell carcinoma, Radiotherapy, Chemotherapy, Elderly patients

## Abstract

**Background:**

Head-and-neck squamous cell carcinoma (HNSCC) is one of the most common malignancies globally, and the number of elderly patients diagnosed with HNSCC is increasing. However, as elderly HNSCC patients are underrepresented in clinical trials, current clinical decision making for this cohort largely lacks clinical evidence.

**Methods:**

Elderly patients (≥65 years) with HNSCC undergoing (chemo)radiotherapy from 2010 to 2018 at Freiburg University Medical Center were assessed for patterns of care, locoregional control (LRC), progression-free (PFS) and overall survival (OS) regarding definitive and adjuvant treatments. Acute and late therapy-associated toxicities were quantified according to CTCAE v5.0.

**Results:**

Two hundred forty-six patients were included in this analysis, of whom 166 received definitive and 80 adjuvant treatment. Two-year rates for OS, PFS and LRC were 56.9, 44.9 and 75.5%, respectively. Survival differed significantly between age groups with an OS of 40 and 22 months and a PFS of 23 and 12 months for patients aged 65–74 or ≥ 75 years, respectively (*p* < 0.05). Concomitant chemotherapy resulted in improved OS in patients aged 65–74 years compared to radiotherapy alone (*p* < 0.05) for definitive treatments, while patients ≥75 years did not benefit (*p* = 0.904). For adjuvant chemoradiotherapy, a trend towards superior OS rates was observed for patients aged 65–74 years (*p* = 0.151). Low performance status (HR = 2.584, 95% CI 1.561–4.274; *p* < 0.001) and smoking (HR = 1.960, 95% CI 1.109–3.464, *p* < 0.05) were the strongest independent prognostic factor in the multivariate analysis for decreased OS. One hundred thirty-eight patients (56.1%) experienced acute grade 3/4 and 45 patients (19.9%) chronic grade 3 toxicities.

**Conclusion:**

Radiotherapy is a feasible treatment modality for elderly HNSCC patients. The relatively low OS compared to high LRC may reflect age and comorbidities. Concomitant chemotherapy should be critically discussed in elderly HNSCC patients.

## Introduction

Head-and-neck squamous cell carcinoma (HNSCC) is a common malignancy and causes more than 300,000 deaths per year worldwide [1]. Twenty-five percent of HNSCC patients are older than 70 years at the time of diagnosis, and this percentage will further increase in Western countries due to ongoing demographic trends [2]. The incidence of patients diagnosed with HNSCC among the elderly is assumed to increase by more than 60% in the Western world by 2030 [3]. As elderly patients were excluded or underrepresented in the landmark trials investigating the role of radiotherapy for HNSCC, extrapolation of trial data to this distinct patient cohort is challenging [4–7].

There is no common definition of elderly patients, and the minimum age for the classification of an elderly patient varies between 65 and 70 years. Based on the consensus definition of the United States National Institute of Aging, elderly patients are subdivided into “young old” (65–74 years), “older old” (75–84 years) and “oldest old” (≥ 85 years) [8]. However, regarding the relation between age and treatment outcome in the oncologic sector, it is generally accepted that the chronological age is often of less importance than the biological age [2].

Several demographic studies have shown that the probability of elderly HNSCC patients to receive curative treatments is considerably lower than for younger patients [9, 10]. For instance, while almost 90% of patients between 45 and 60 years receive standard treatment, only about 60% of patients exceeding 70 years are treated according to medical guidelines [9]. Even with equal comorbidity index values, age has been established as an independent factor for non-standard treatment [9]. Regarding patient priorities, it has been shown that, compared to younger patients, elderly cancer patients focused more on the quality of life than an overall survival benefit [11]. However, quality of life in elderly patients receiving curative HNSCC treatment has been reported to be similar to younger patients in retrospective datasets [12].

Comorbidities are significantly more present in elderly HNSCC patients, thus complicating both surgical approaches and concomitant chemoradiotherapy in this cohort. Previous meta-analyses have demonstrated that the benefit of concomitant chemotherapy decreases with age [13]. Considering the significantly higher burden of comorbidities, elderly patients are more likely to die from non-cancer deaths; for instance, the percentage of deaths not due to HNSCC was 39% for patients older than 70 years in a large meta-analysis [13]. Similarly, compared to conventional radiotherapy, altered fractionation showed less advantage in elderly patients than in younger patients [14].

In this study, we analyzed demographic, outcome and toxicity data in a large single-center cohort of 246 elderly patients receiving (chemo)radiotherapy for HNSCC between 2010 and 2018. Additionally, we investigated risk factors correlating with decreased treatment response in these patients.

## Material and methods

### Patients and treatment

This retrospective single-center analysis enrolled all patients older than 65 years treated with radiotherapy or chemoradiation for histologically confirmed HNSCC between 2010 and 2018 at the Department of Radiation Oncology, University of Freiburg Medical Center. The study was approved in advance by the institutional ethical review committee (reference no. 551/18). Demographic and clinical patient data were retrospectively taken from electronic patient records, and pathological data were extracted from the pathology reports. A positive smoking status referred to a smoking history of at least 10 years. Staging of HNSCC was based on the 7th Edition of the UICC TNM classification.

Treatment was based on multidisciplinary tumor board recommendations. For treatment planning and application, patients were immobilized with individually moulded thermoplastic masks. Radiotherapy planning was conducted with Oncentra MasterPlan® (Nucletron BV, Veenendaal, The Netherlands) and Eclipse™ planning softwares (Varian Medical Systems) (Additional file 1: Figure S1). Depending on the time period of treatment, conformal 3-dimensional (3DRT) or intensity-modulated radiotherapy (IMRT) was used for treatment, and radiotherapy was administered in 2 Gy fractions to a total dose of 70 Gy for definitive and 60–66 Gy for adjuvant treatments. For clinical target volume (CTV) delineation, a margin of 0.5–1 cm was added to the gross tumor volume (GTV) and adjusted for anatomic compartments. The CTV was expanded using a margin of 0.5–0.7 cm in order to obtain the planning target volume (PTV). Elective lymph nodes were treated with 50–54 Gy. In dependence of the primary tumor localization, cervical lymph node regions were included for elective node irradiation based on the ESTRO consensus guidelines [15].

Concurrent chemotherapy was scheduled for all patients undergoing definitive treatment and in case of incomplete resection (R1/R2) or extranodal extension (ENE) in the adjuvant setting. All systemic agents were administered as intravenous infusions in an inpatient setting. Chemotherapy was defined as completed for all patients receiving a cumulative dose of ≥200 mg/m^2^ cisplatin or ≥ 450 mg/m^2^ carboplatin.

### Survival and toxicity

Patients presented for follow-up examinations in 3-monthly intervals in the first year and 6-monthly intervals from year 2. Follow-up visits included a physical examination and imaging by CT or MRI of the head-and-neck region. In case of clinical evidence for loco-regional recurrence or distant metastases, additional tests or imaging modalities were carried out at the discretion of the treating physician. Overall survival (OS) was calculated from the completion of treatment to death from any cause, and progression-free survival (PFS) was assessed as the interval between treatment completion and disease progression at any site or death of any cause. Locoregional control (LRC) was defined as the absence of any progression of the primary tumor or the onset or progression of any cervical lymph node metastases. Missing survival data were acquired from the record sections of the federal state authorities of Baden-Württemberg via the Comprehensive Cancer Center Freiburg. Acute and chronic toxicities were classified based on the CTCAE v. 5.0.

### Statistical analyses

Actuarial OS, PFS and LRC rates were analyzed using the Kaplan-Meier method with the log-rank test to evaluate statistical significance. Univariate and multivariate analyses were performed using the Cox proportional hazards model. *P*-values below 0.05 were considered statistically significant. All statistical analyses were carried out using IBM SPSS Statistics software version 25 (IBM, Armonk, NY, USA).

## Results

### Patient and treatment characteristics

A total of 246 patients with histologically confirmed HNSCC were included in this analysis. Patients were predominantly male (69.1%), and median age was 72 years (65 to 96 years). 153 (62.2%) of our patients were younger than 75 years, therefore classified as “young olds”, and 93 (37.8%) were 75 years or older, defined as “older olds/oldest olds” according to the consensus definition of the United States National Institute of Aging. Tumor sites affected the oropharynx (*n* = 79, 32.1%), oral cavity (*n* = 57, 23.2%), larynx (*n* = 41, 16.7%) and hypopharynx (*n* = 29, 11.8%). Multi-level tumors (*n* = 15, 6.1%), parotid (*n* = 6, 2.4%), nasopharyngeal tumors (*n* = 4, 1.6%) and tumors of other salivary glands (*n* = 3, 1.2%) were rare. Other tumor sites (*n* = 12, 4.9%) included the nasal cavity or paranasal sinuses.

The majority of tumors were first diagnosed in locoregionally advanced stages with 150 patients (61.0%) of patients suffering from a T3/4 tumor and 164 patients (66.7%) exhibiting a lymphatic spread. Only 10 patients (4.1%) had distant metastasis at time of initial diagnosis. With all tumors being of squamous cell origin, most tumors were graded G2 (*n* = 157, 63.8%) or G3 (*n* = 74, 30.1%). HPV was detected in 34 patients (41.0% of HPV-tested tumors) but was not assessed in the majority of patients (*n* = 163, 66.3%). For oropharyngeal carcinoma, HPV status was assessed in 43 patients (54.4% of oropharyngeal carcinoma) and was positive in 55.8% (*n* = 24) of analyzed oropharyngeal tumor samples. One hundred forty-two patients were smokers (57.7%), and the majority of analyzed elderly patients were in a generally good condition with 55.3% of patients (*n* = 136) having a Karnofsky performance status scale of 90% or 100%. Detailed patient characteristics can be found in Table 1 and Additional file 4: Table S1.

**Table 1:** Patient characteristics consisting elderly HNSCC patients treated by (chemo)radiotherapy in our institution between 2010 and 2018 (*n* = 246). Staging of HNSCC was based on the 7th Edition of the UICC TNM classification

		n	%
Sex	male	170	69.1
	female	76	30.9
Age	65–74 years	153	62.2
	≥ 75 years	93	37.8
Smoking	non-smoker	54	22.0
	smoker	142	57.7
	missing	50	20.3
Karnofsky	100%	28	11.4
	90%	108	43.9
	80%	51	20.7
	70%	23	9.3
	60%	14	5.7
	50%	3	1.2
	40%	1	0.4
	missing	18	7.3
Localization	nasopharynx	4	1.6
	oropharynx	79	32.1
	hypopharynx	29	11.8
	oral cavity	57	23.2
	larynx	41	16.7
	parotid glands	6	2.4
	other salivary glands	3	1.2
	multi-level	15	6.1
	others	12	4.9
T-stage	T1	35	14.2
	T2	53	21.5
	T3	64	26.0
	T4	86	35.0
N-stage	N0	82	33.3
	N1	33	13.4
	N2	120	48.8
	N3	11	4.5
M-stage	M0	232	94.3
	M1	10	4.1
UICC	I	24	9.8
	II	20	8.1
	III	48	19.5
	IVA/B	145	58.9
	IVC	9	3.7
Grading	G1	6	2.4
	G2	157	63.8
	G3	74	30.1
	G4	1	0.4
HPV	HPV-negative	49	19.9
	HPV-positive	34	13.8
	missing	163	66.3

One hundred sixty-six patients (67.5%) underwent definitive (chemo-)radiation, and 80 patients (32.5%) were postoperatively treated (Table 2). Overall therapy adherence was very high with 86.6% of patients (*n* = 213) completing the planned radiotherapy. This resulted in a mean dose of 65.4 Gy in the primary therapy cohort and 60.2 Gy in the adjuvant cohort.

**Table 2:** Treatment details for (chemo)radiotherapy of elderly HNSCC patients (*n* = 246). Chemotherapy completion was assumed, if patients received at least 200 mg/m^2^ cisplatin or 450 mg/m^2^ carboplatin

Radiation therapy	n	%
completed	213	86.6
discontinued	33	13.4
definitive	166	
completed	141	84.9
adjuvant	80	
completed	72	90.0
adjuvant		
mean radiation dose	60.2 Gy	
mean single dose	2.0 Gy	
definitive		
mean radiation dose	65.4 Gy	
mean single dose	2.0 Gy	
Chemotherapy	n	%
planned	147	
completed	109	74.1

Concomitant systemic treatment was administered in 147 patients (59.8%), and almost all patients received platinum-based treatment with only 3 patients treated with concomitant mitomycin C, cetuximab or nivolumab. One hundred fourteen patients (68.7%) receiving definitive treatment were treated with chemotherapy, while 33 patients with adjuvant treatment received concurrent chemotherapy (41.3%). Completion of concomitant chemotherapy, defined as more than 200 mg/m^2^ cisplatin or more than 450 mg/m^2^ carboplatin, could be achieved in 109 patients, resulting in a completion rate of 74.1%.

### Treatment outcome

**Fig. 1:**
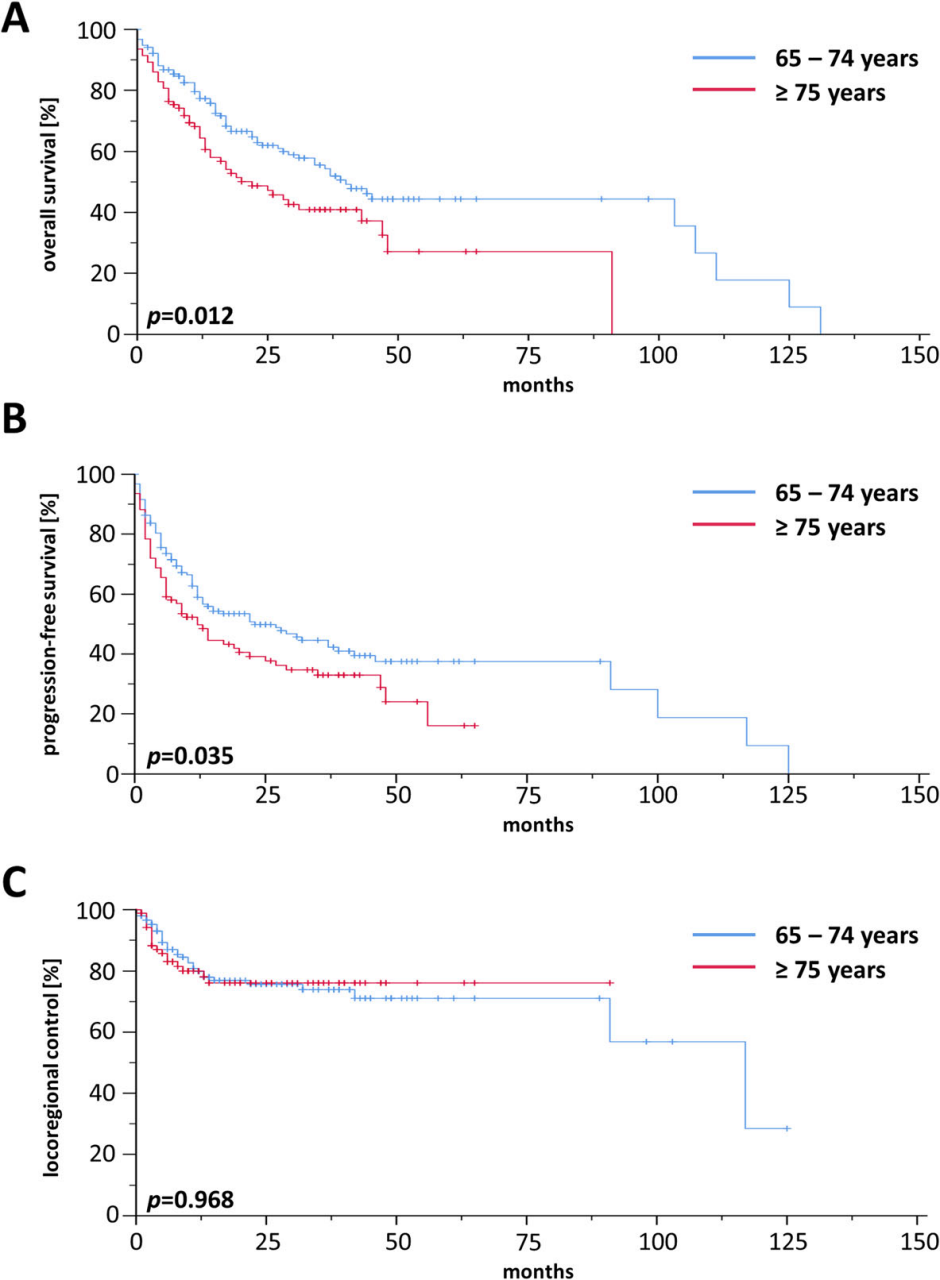
Kaplan-Meier curves for OS (**a**), PFS (**b**) and LRC (**c**) of elderly HNSCC patients treated by definitive or adjuvant (chemo)radiotherapy (*n* = 246). *P*-values of log-rank-tests are shown

**Fig. 2:**
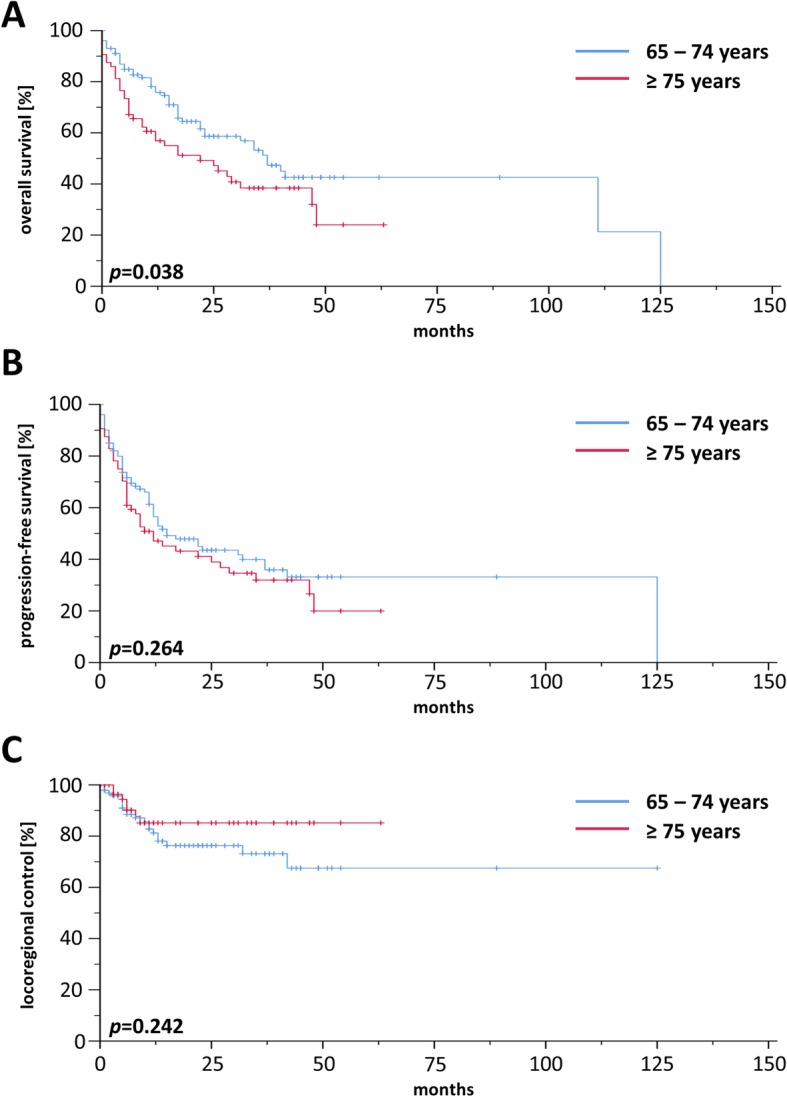
Kaplan-Meier curves showing OS (**a**), PFS (**b**) and LRC (**c**) of elderly HNSCC patients receiving definitive (chemo)radiotherapy (*n* = 166)

**Fig. 3:**
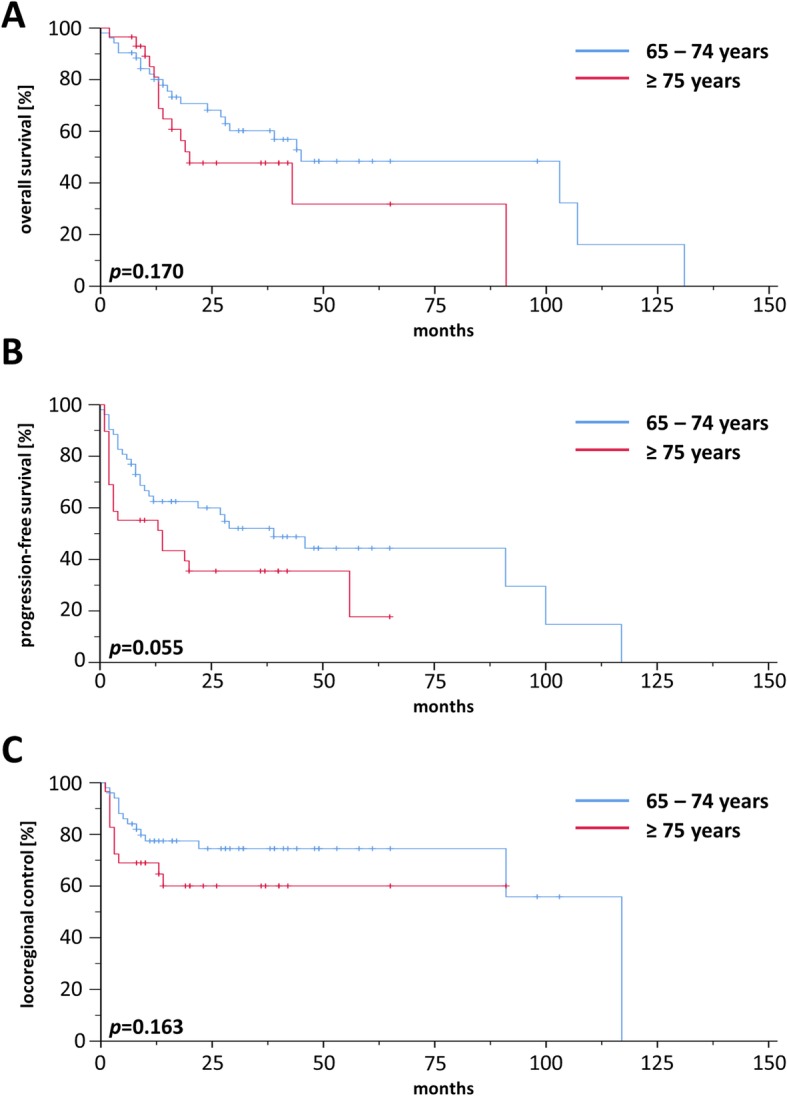
OS (**a**), PFS (**b**) and LRC (**c**) of HNSCC patients ≥65 years who have received adjuvant (chemo)radiotherapy (*n* = 80)

**Fig. 4:**
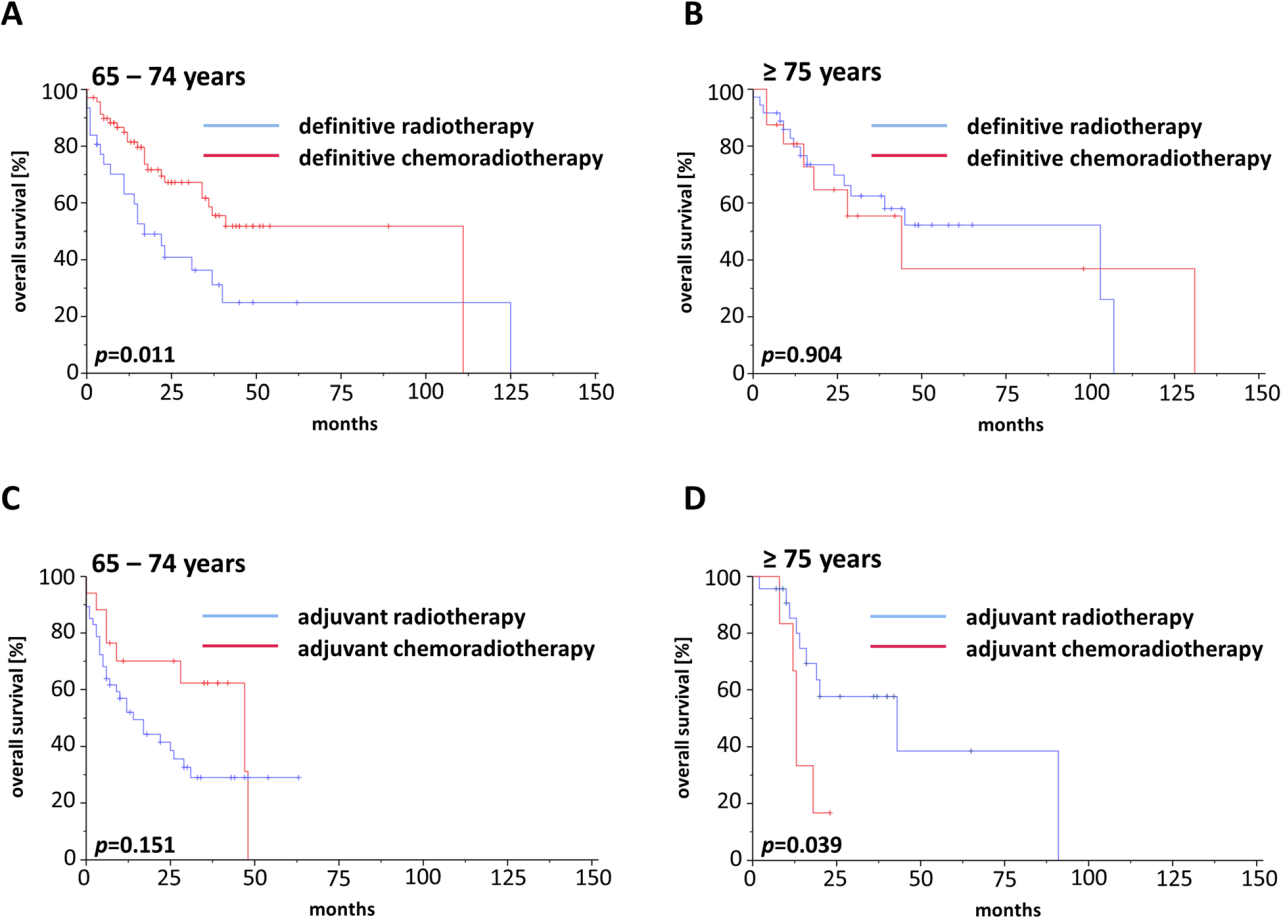
Kaplan-Meier curves demonstrating OS of HNSCC patients treated by radiotherapy (blue line) or chemoradiotherapy (red line). **a**, **b** Elderly HNSCC patients aged 65–74 years (**a**) or ≥ 75 years (**b**) with definitive treatment. **c**, **d** Elderly HNSCC patients with 65–74 years (**c**) or ≥ 75 years (**d**) treated in an adjuvant setting

For the whole cohort, 2-year rates for OS, PFS and LRC were 56.9, 44.9 and 75.5%, respectively, and median OS, PFS and LRC ranged at 34.0, 17.0, and 117.0 months, respectively (Additional file 2: Figure S2). Comparisons between patients treated by definitive or adjuvant (chemo)radiotherapy did not reveal a significant OS difference in our elderly HNSCC patient cohort (*p* = 0.165) (Additional file 3: Figure S3). Survival differed considerably between “young olds” (65–74 years) and “older/oldest olds” (≥ 75 years) with a median OS of 40 vs. 22 months (*p* < 0.05, log-rank test) (Fig. 1a) and a median PFS of 23 vs. 12 months (*p* < 0.05) (Fig. 1b), while LRC did not significantly vary between age groups (*p* = 0.968) (Fig. 1c). Survival analyses of the patient cohort with definitive treatment revealed a survival benefit for patients aged 65–74 years (Fig. 2a) in terms of OS (*p* < 0.05) with no differences regarding PFS (Fig. 2b) or LRC (Fig. 2c). In contrast, there were no differences for OS, PFS and LRC between “young olds” (65–74 years) and “older/oldest olds” (≥ 75 years) when analysis was limited to patients with adjuvant (chemo)radiotherapy (Fig. 3). Completion of concomitant chemotherapy as part of definitive chemoradiotherapy resulted in improved OS in patients aged 65–74 years compared to radiotherapy alone (*p* < 0.05), while chemotherapy had no measurable benefit in patients above 75 years (*p* = 0.904) (Fig. 4a-b). For adjuvant radiotherapy, a trend towards superior OS rates were observed for patients aged 65–74 years treated by chemoradiotherapy in comparison with radiotherapy alone, although statistical significance was not reached (*p* = 0.151) (Fig. 4c). Considering the limited patient number of patients above 75 years undergoing adjuvant treatment that prohibited any conclusive analysis, we observed a statistically significant inferiority of chemoradiotherapy in terms of OS when compared to radiotherapy (*p* < 0.05) (Fig. 4d). Chemotherapy prescription did not influence the likelihood of radiotherapy completion in our cohort (*p* = 0.972, χ2 test).

On univariate analysis, an age of 75 years and older (HR = 1.584, 95% CI 1.101–2.280, *p* < 0.05), smoking history (HR = 1.731, 95% CI 1.045–2.868, *p* < 0.05), impaired renal function as defined by an estimated glomerular filtration rate below 60 ml/min/1.73 m^2^ at treatment initiation (HR = 1.537, 95% CI 1.024–2.308, *p* < 0.05), non-completion of prescribed chemotherapy (HR = 1.481, 95% CI 1.025–2.140, *p* < 0.05) and low Karnofsky performance status (HR = 2.803, 95% CI 1.817–4.324, *p* < 0.001) were found to have a significant prognostic value regarding reduced OS (Table 3). On multivariate Cox regression analysis of these parameters, only low performance status (HR = 2.584; 95% CI 1.561–4.274; *p* < 0.001) and smoking history (HR = 1.960; 95% CI 1.109–3.464; *p* < 0.05) were found to significantly impair OS. In the multivariate analysis, there was a statistically non-significant trend towards reduced OS for patients aged 75 years and older (HR = 1.564, 95% CI 0.962–2.543, *p* = 0.072).

**Table 3:** Univariate and multivariate analysis of clinical parameters regarding OS in elderly HNSCC patients receiving radiotherapy or chemoradiotherapy

Parameter	HR	CI 95%	*p*-value
Univariate			
Age ≥ 75 / 65–74 years	1.584	1.101–2.280	0.013
Smoker / non-smoker	1.731	1.045–2.868	0.033
GFR < 60 / ≥ 60 ml/min/1.73m^2^	1.537	1.024–2.308	0.038
Chemotherapy non-completed / completed	1.481	1.025–2.140	0.037
Karnofsky < 80 / ≥ 80%	2.803	1.817–4.324	< 0.001
Multivariate			
Age ≥ 75 / 65–74 years	1.564	0.962–2.543	0.072
Smoker / non-smoker	1.960	1.109–3.464	0.020
GFR < 60 / ≥ 60 ml/min/1.73m^2^	1.047	0.612–1.791	0.868
Chemotherapy non-completed / completed	0.852	0.736–1.174	0.501
Karnofsky < 80 / ≥ 80%	2.584	1.561–4.274	< 0.001

### Toxicity

Moderate to severe acute toxicity was found to be relatively high in our elderly patient cohort, with 56.1% of patients (*n* = 138) suffering from at least one acute CTCAE grade 3/4 toxicity (Additional file 5: Table S2). No acute grade 5 toxicity was observed in our cohort. Most prevalent grade 3/4 toxicities were dysphagia, cytopenia and mucositis with 80 (32.5%), 69 (28.0%) and 46 (18.7%) affected patients, respectively (Additional file 6: Table S3).

Chronic toxicities were generally mild but widespread, with 66.4% of patients (*n* = 150) exhibiting a grade 1/2 toxicity. Forty-five patients (19.9%) developed a grade 3 chronic toxicity, most commonly dysphagia, jaw and dental injuries as well as pain with 31, 10 and 4 events, respectively. However, no chronic grade 4 or 5 toxicities were observed. Overall, xerostomia, dysgeusia, dysphagia and pain were most frequent among chronic toxicities.

## Discussion

Here, we demonstrated in a large single-center cohort that radiotherapy and chemoradiotherapy are feasible treatment modalities also for elderly patients with HNSCC and are associated with relatively high LRC rates. However, with a median OS of 34 months, oncological results for this elderly cohort are worse than for the highly selected younger patient cohorts in several large randomized controlled trials. For example, postoperative chemoradiotherapy resulted in median overall survival rates of 44.9 months in the RTOG 9501 trial and 72 months in the EORTC 22931 trial [5, 6]. In the older Intergroup-126 trial, median OS for cisplatin-based chemoradiotherapy was calculated at 19.1 months, while in a more recent cohort study employing IMRT techniques, OS ranged above 60 months for most tumor subsites [4, 16].

Similarly to the results of other retrospective studies about (chemo)radiotherapy for elderly HNSCC patients, baseline performance status was found to be a key prognostic factor for OS in our cohort both in the univariate and the multivariate analysis [17, 18]. Since prediction of a patient’s performance status has a high interobserver variability, geriatric assessments may be used as an alternative. Geriatric assessments are able to access many different domains of life of elderly patients including their functional, nutritional, cognitive, psychosocial and socioeconomic status [19]. However, no geriatric score could be calculated due to the retrospective nature of our analysis. Studies have demonstrated that risk models based on a geriatric assessment are appropriate methods for predicting chemotherapy toxicity [20, 21]. As comprehensive multidisciplinary geriatric assessments may be challenging to conduct in the daily routine, screening tools such as the G8 have been evaluated to identify elderly HNSCC patients who would require in-depth geriatric assessments [22].

A positive smoking status significantly decreased the survival of elderly HNSCC patients both in the univariate and multivariate analysis. Previous studies have shown that continuous smoking of HNSCC patients is associated with more severe treatment-related toxicities, higher risk for developing a second primary tumor and worse outcomes in terms of cancer-specific survival [23–25]. Additionally, a higher percentage of HPV-positive tumors in the cohort of non-smoking patients could also contribute to the observed survival benefit for elderly non-smokers, and it is conceivable that a positive smoking history is also linked to an increased non-cancer-related mortality. Following international HNSCC treatment guidelines, smoking cessation should be encouraged and supported by physicians treating HNSCC patients [26]. Unfortunately, many oncologists only access the smoking status but do not routinely offer smoking cessation support [27].

As an impaired renal function is known to be an independent prognosticator for many cancer entities such as gynecological and hematological malignancies and may also influence the ability to tolerate systemic treatments, we investigated the influence of the glomerular filtration rate on the survival of elderly HNSCC patients [28]. Reduced glomerular filtration rates were found to negatively influence the survival only in the univariate but not in the multivariate analysis. These observations are in line with a previous study investigating the impact of the renal function on the survival of patients with different malignancies [28]. Other laboratory parameters such as pre-treatment serum CRP levels have also been shown to predict prognosis of patients with HNSCC treated by definitive radiotherapy [29]. As CRP serum levels were not available for a significant number of patients in our study cohort, we did not include this marker in our Cox regression analysis; however, further studies evaluating the role of established laboratory parameters regarding survival prediction of elderly HNSCC patients undergoing radiotherapy are warranted.

Beyond patient performance, age was found to be a prognostic factor for OS in our cohort in the univariate, but not the multivariate analysis. There are many retrospective studies showing that the survival among older patients treated by radiotherapy alone is comparable to that of younger patients [30, 31]. In an older meta-analysis by Pignon and colleagues summarizing individual data from 1589 HNSCC patients receiving radiotherapy or chemoradiotherapy, survival and treatment-related toxicities did not differ between younger and older patients [32]. However, the available data contained only a small percentage of elderly patients and did not include patients aged 75 years or older. Our findings regarding an age-dependent outcome in elderly HNSCC patients seem plausible regarding the potential influence of co-morbidities and the overall life expectancy of this cohort. Previous datasets have shown up to 40% non-cancer-related deaths in HNSCC patients above 70 years [13].

The use and benefit of the addition of chemotherapy to radiotherapy in elderly patients has been subject to debate. In a large meta-analysis, the additional effect of concomitant chemotherapy was found age-dependent with patients below 60 years benefitting most and patients above 70 having no measurable benefit [13]. In this analysis, the majority of patients received platinum-based chemoradiotherapy; however, considering the toxicity profile of platinum compounds, many elderly patients may not qualify for platinum-based chemotherapy due to renal, cardiac or other comorbidities. Therefore, epidermal growth factor receptor (EGFR) inhibitors have been suggested as alternative systemic agents that may provide a suitable alternative for unfit elderly patients. Addition of the EGFR inhibitor cetuximab has demonstrated superiority over radiotherapy alone in terms of LRC and OS for locoregionally advanced HNSCC [7]. However, the vast majority of patients enrolled in this trial were below 70 years, had a good performance status and did not exhibit significant comorbidities [7]. Post-hoc analyses revealed that elderly patients were less likely to benefit from radiotherapy plus cetuximab [33]. Additionally, recent data from the De-ESCALaTE and RTOG 1016 trials comparing concomitant cetuximab with cisplatin during radiotherapy in HPV-positive oropharyngeal carcinoma demonstrated inferiority of the EGFR inhibitor in terms of OS and PFS, and it remains to be demonstrated if these results also hold true for other HNSCC subtypes [34, 35]. Recently, a small study investigated the role of hypofractionated radiotherapy in combination with cetuximab for locoregionally advanced HNSCC in vulnerable elderly patients and reported insufficient treatment responses with disease progression in half of the patients after 3 months and high toxicity rates, concluding that cetuximab-based hypofractionated radiotherapy is no alternative for elderly HNSCC patients [36]. This has to be born in mind when considering the relatively high rates of acute toxicities that may transiently but significantly impact the quality of life of elderly patients undergoing standard (chemo)radiotherapy.

In general, we did not detect an OS difference between elderly HNSCC patients treated by definitive or adjuvant (chemo)radiotherapy. Interestingly, addition of chemotherapy resulted in a survival benefit for patients aged 65–74 years in the definitive but not in the adjuvant treatment cohort. For patients aged 75 years or older, adjuvant chemoradiotherapy was found to be inferior regarding OS compared to adjuvant radiotherapy highlighting the importance of a R0 resection in elderly HNSCC patients to avoid postoperative chemoradiotherapy. Therefore, pre-treatment patient selection especially in elderly HNSCC patients aged 75 years or older is immensely important in order to detect patients who have a high probability to exhibit postoperative risk features such as positive resection margins or ENE. As both landmark trials that established the relevance of postoperative chemoradiotherapy for positive resection margins and ENE either excluded patients above 70 years (EORTC 22931 trial) or enrolled only a small minority of patients older than 70 years (RTOG 9501 trial), the evidence for postoperative chemoradiotherapy in “older/oldest olds” HNSCC patients is limited [5, 6]. Recently, a large retrospective analysis with 1199 patients aged 70 years or above demonstrated that postoperative chemoradiotherapy resulted in an improved survival with 3-year OS rates of 52.4% after chemoradiotherapy and 43.4% after radiotherapy alone [37]. However, the survival benefit was limited to patients with advanced nodal status, and patients with N0 status had a trend towards impaired OS after chemoradiotherapy compared to radiation alone. As almost 50% of the patients were between 70 and 74 years old in this study, the conflicting results may be explained by the different age subdivision applied in our study between “young olds” (65–74 years) and “older/oldest olds” (≥ 75 years) based on the consensus definition of the United States National Institute of Aging. Another large retrospective study with 1686 patients from the National Cancer Database (NCDB) using a propensity score matching did not find a survival benefit for chemoradiation in elderly HNSCC patients with positive resection margins or ENE [38]. While the study of Yoshida et al. included cases of HNSCC diagnosed between 2004 and 2013, Giacalone and colleagues examined HNSCC cases between 1998 and 2011 which may explain the different results of both studies [37, 38]. Additionally, another large retrospective analysis of 10,599 patients from the SEER database observed no benefit of adding chemotherapy to radiotherapy for elderly HNSCC patients [39]. Considering these discrepancies, additional large multi-center analyses will be needed to elucidate the role of adjuvant concurrent chemoradiotherapy in HNSCC patients above 70 years.

Overall, the relatively high acute toxicity rate in our study population with 56.1% of patients reporting about acute CTCAE grade 3/4 toxicities should be considered when treating elderly HNSCC patients. In another retrospective study investigating the results of chemoradiotherapy for elderly patients with locally advanced HNSCC, dysphagia was a common side effect with 62% of patients requiring gastrostomy tube feeding [40]. While almost 10% of patients suffered from grade 4 mucositis in the study of Maggiore and colleagues, we did not observe any grade 4 mucositis in our cohort [40]. Interestingly, von der Grün and colleagues reported comparable toxicity rates for mucositis, dermatitis, dysphagia and pain between patients younger than 65 years and patients aged 65 years or older [17]. In order to access treatment-related toxicities more precisely, prospective trials regarding radiotherapy of elderly HNSCC patients with a focus on treatment-induced adverse reactions and resulting quality of life are required.

While our analysis provides outcome data in a large homogenous cohort of elderly HNSCC patients undergoing radiotherapy, it has limitations due to its retrospective character. Patient characteristics such as detailed comorbidities and quality of live as well as in-depth biological tumor characteristics such as p16 status could not be provided for all patients included in this study. The impact of comorbidities on the survival of elderly HNSCC patients has been shown previously, and several comorbidity scores like the Charlson Comorbidity Index or the Adult Comorbidity Evaluation-27 score have been shown to be independent predictors of survival [41, 42]. P16 or HPV as markers for an improved outcome in a subset of oropharyngeal cancer patients were not routinely examined in our patients, and it needs to be considered that HPV may influence the outcome also of elderly patients as reported for the general cohort of patients with HPV-positive oropharyngeal cancers [43, 44].

In summary, our large single-center analysis consisting of 246 elderly HNSCC patients indicates that radiotherapy leads to respectable LRC but relatively low OS rates and significant acute toxicities. Additionally, only patients below 75 years benefitted from concomitant chemotherapy as part of definitive chemoradiotherapy. While these findings may bear relevance for treatment decisions in elderly patients, prospective trials are needed to further corroborate our findings and to find the optimal treatment modalities for this distinct patient cohort.

## Supplementary information

**Additional file 1: Figure S1.** Definitive radiotherapy for a HNSCC in a 79-year-old patient. A cT3 cN3 M0 oral cavity carcinoma was treated with a simultaneous integrated boost-intensity-modulated radiotherapy (SIB-IMRT). High-risk PTV was treated with 69.3 Gy delivered in 33 fractions, while low-risk PTV received 56.1 Gy in 33 fractions. (**A**) CT image showing a locally advanced oral cavity carcinoma with bilateral cervical lymph node metastases. (**B**) Pretherapeutic T1-weighted MRI scan showing bilateral cervical lymph node metastases with central necrosis. (**C**, **D** and **E**) SIB-IMRT plan demonstrating the dose distribution in an axial (**C**), coronary (**D**) and sagittal (**E**) scan image.

**Additional file 2: Figure S2.** OS (**A**), PFS (**B**), LRC (**C**) of the complete patient cohort consisting elderly HNSCC patients treated by (chemo)radiotherapy in our institution between 2010 and 2018 (*n* = 246).

**Additional file 3: Figure S3.** Kaplan-Meier curves showing OS of elderly HNSCC patients treated by definitive (chemo)radiotherapy (blue line) or adjuvant (chemo)radiotherapy (red line).

**Additional file 4: Table S1.** Patient characteristics consisting elderly HNSCC patients treated by (chemo)radiotherapy in our institution separated by different age groups.

**Additional file 5: Table S2.** Toxicity results after (chemo)radiotherapy of elderly patients with HNSCC according to the Common Terminology Criteria for Adverse Events (CTCAE) v5.0.

**Additional file 6: Table S3.** Toxicity results consisting various (chemo)radiotherapy-related adverse reactions according to the Common Terminology Criteria for Adverse Events (CTCAE) v5.0.

## Data Availability

The datasets used and analyzed during the current study are available from the corresponding author on reasonable request.

## Abbreviations

3DRT: 3-dimensional conformal radiotherapy
CI: Confidence interval
CRP: C-reactive protein
CTCAE: Common terminology criteria for adverse events
CTV: Clinical target volume
ENE: Extranodal extension
ESTRO: EUROPEAN Society for Radiotherapy and Oncology
GTV: Gross tumor volume
HNSCC: Head and neck squamous cell carcinoma
HPV: Human papilloma virus
HR: Hazard ratio
IMRT: Intensity-modulated radiotherapy
LRC: Loco-regional control
NCDB: National Cancer Database
OS: Overall survival
PFS: Progression-free survival
PTV: Planning target volume
