# Supplementary figures and images for: Radiotherapy for geriatric head-and-neck cancer patients: what is the value of standard treatment in the elderly?
Source: Radiat Oncol. 2020 Feb 4;15:31. doi: 10.1186/s13014-020-1481-z (PMC7001207; doi:10.1186/s13014-020-1481-z)

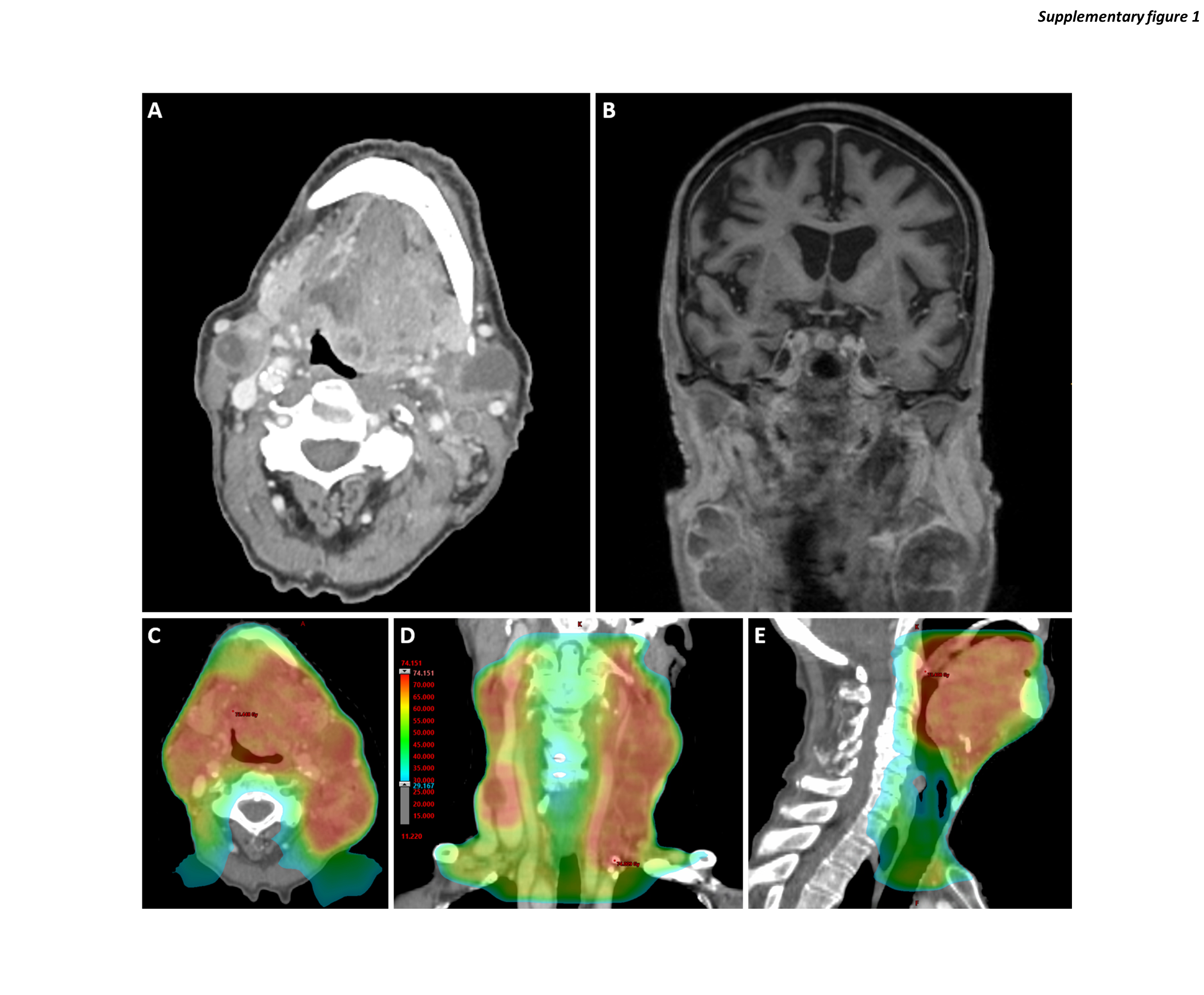

Supplement: Supplementary file 1 — Additional file 1: Figure S1. Definitive radiotherapy for a HNSCC in a 79-year-old patient. A cT3 cN3 M0 oral cavity carcinoma was treated with a simultaneous integrated boost-intensity-modulated radiotherapy (SIB-IMRT). High-risk PTV was treated with 69.3 Gy delivered in 33 fractions, while low-risk PTV received 56.1 Gy in 33 fractions. (A) CT image showing a locally advanced oral cavity carcinoma with bilateral cervical lymph node metastases. (B) Pretherapeutic T1-weighted MRI scan showing bilateral cervical lymph node metastases with central necrosis. (C, D and E) SIB-IMRT plan demonstrating the dose distribution in an axial (C), coronary (D) and sagittal (E) scan image. [file 13014_2020_1481_MOESM1_ESM.tif]

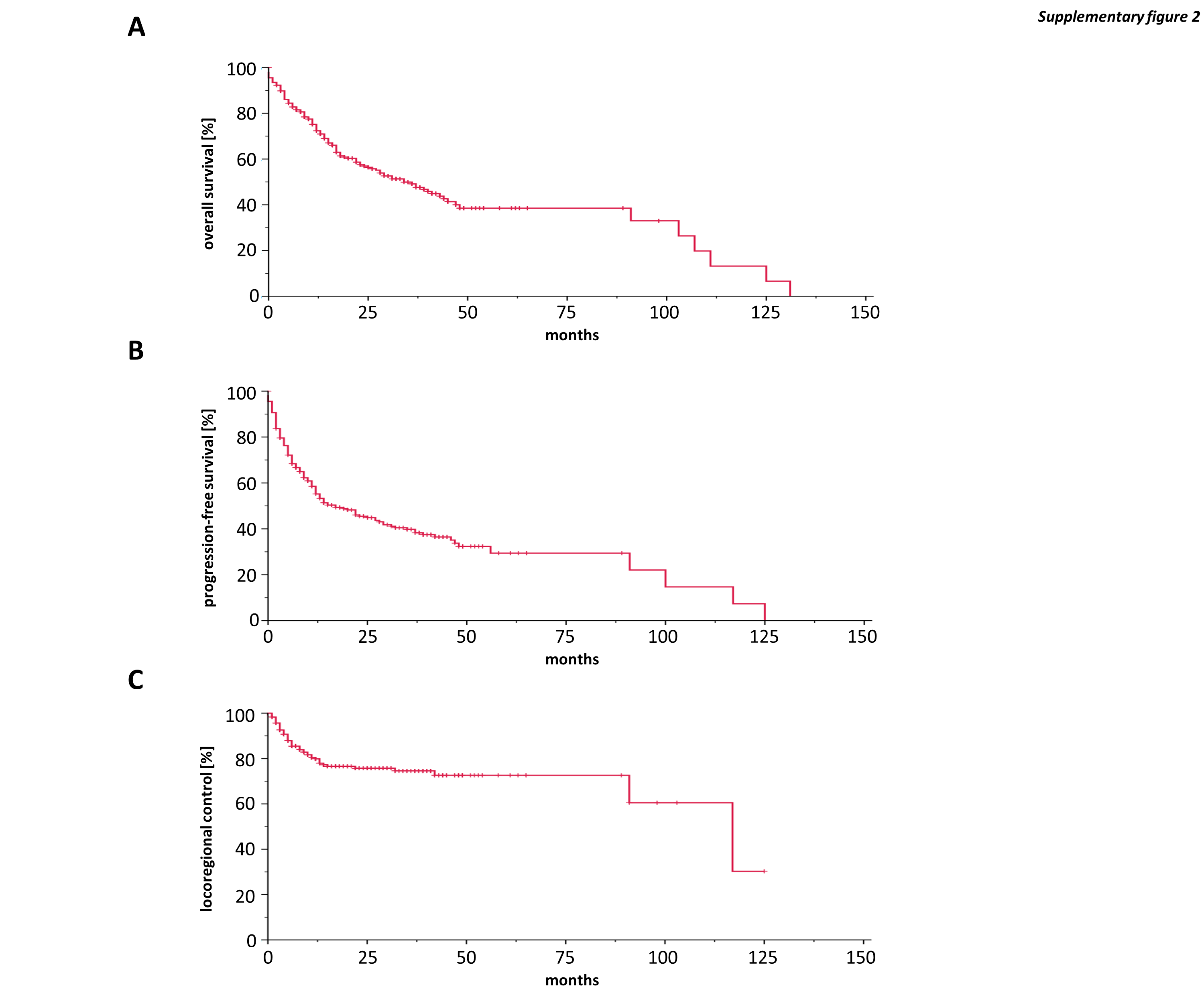

Supplement: Supplementary file 2 — Additional file 2: Figure S2. OS (A), PFS (B), LRC (C) of the complete patient cohort consisting elderly HNSCC patients treated by (chemo)radiotherapy in our institution between 2010 and 2018 (n = 246). [file 13014_2020_1481_MOESM2_ESM.tif]

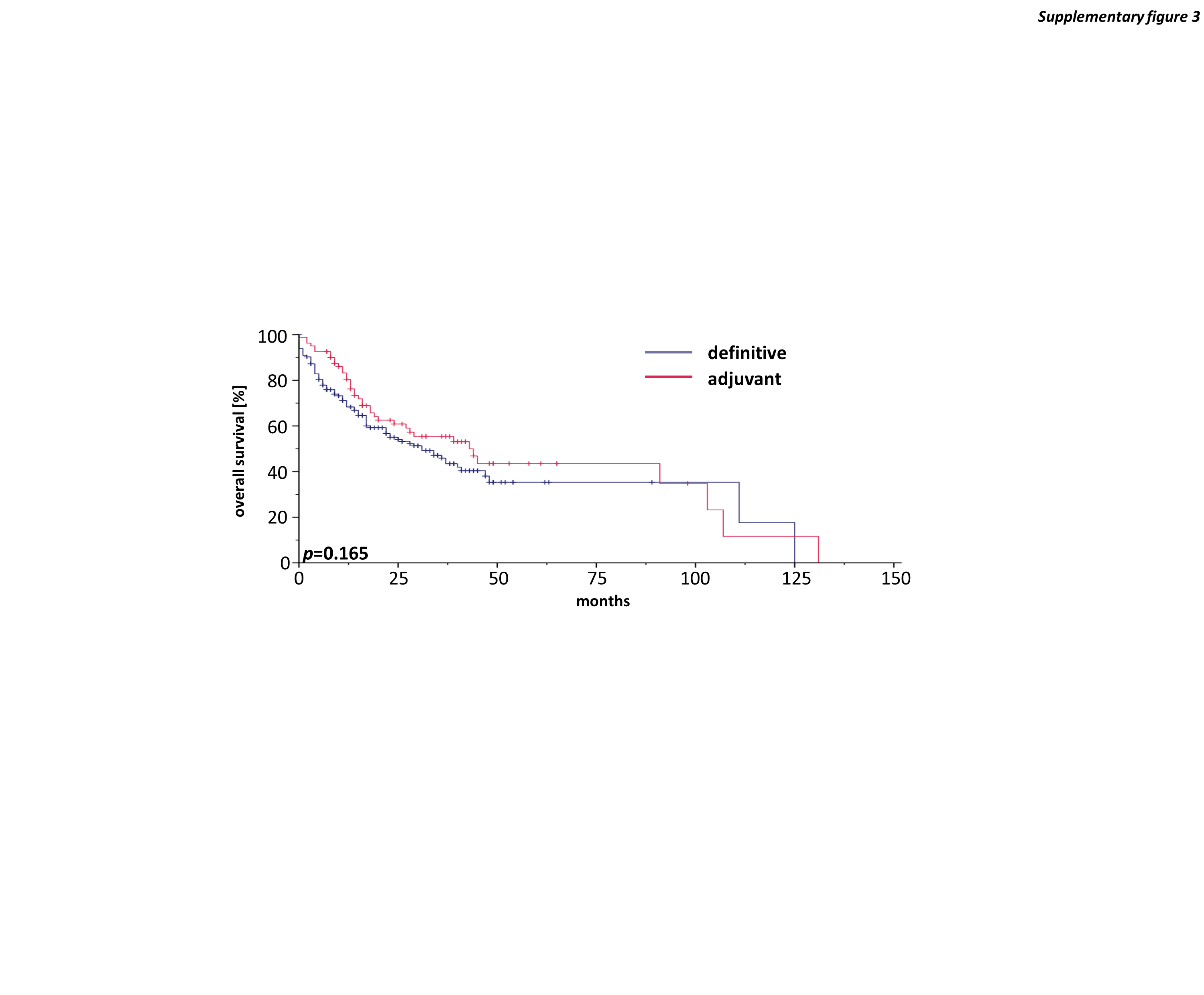

Supplement: Supplementary file 3 — Additional file 3: Figure S3. Kaplan-Meier curves showing OS of elderly HNSCC patients treated by definitive (chemo)radiotherapy (blue line) or adjuvant (chemo)radiotherapy (red line). [file 13014_2020_1481_MOESM3_ESM.tif]
