# Supplementary material for: Radiotherapy for geriatric head-and-neck cancer patients: what is the value of standard treatment in the elderly?
Source: Radiat Oncol. 2020 Feb 4;15:31. doi: 10.1186/s13014-020-1481-z (PMC7001207; doi:10.1186/s13014-020-1481-z)
Supplement: Supplementary file 4 — Additional file 4: Table S1. Patient characteristics consisting elderly HNSCC patients treated by (chemo)radiotherapy in our institution separated by different age groups. [file 13014_2020_1481_MOESM4_ESM.docx]

|  | |  |  | **65 – 74 years** | | **≥75 years** | |
| --- | --- | --- | --- | --- | --- | --- | --- |
|  | | **n** | **%** | **n** | **%** | **n** | **%** |
| **Sex** | male | 170 | 69.1 | 108 | 70.6 | 62 | 66.7 |
|  | female | 76 | 30.9 | 45 | 29.4 | 31 | 33.3 |
| **Smoking** | non-smoker | 54 | 22.0 | 26 | 17.0 | 28 | 30.1 |
|  | smoker | 142 | 57.7 | 100 | 65.4 | 42 | 45.2 |
|  | missing | 50 | 20.3 | 27 | 17.6 | 23 | 24.7 |
| **Karnofsky** | 100% | 28 | 11.4 | 20 | 13.1 | 8 | 8.6 |
|  | 90% | 108 | 43.9 | 71 | 46.4 | 37 | 39.8 |
|  | 80% | 51 | 20.7 | 32 | 21.0 | 19 | 20.4 |
|  | 70% | 23 | 9.3 | 12 | 7.8 | 11 | 11.8 |
|  | 60% | 14 | 5.7 | 6 | 3.9 | 8 | 8.6 |
|  | 50% | 3 | 1.2 | 2 | 1.3 | 1 | 1.1 |
|  | 40% | 1 | 0.4 | 0 | 0.0 | 1 | 1.1 |
|  | missing | 18 | 7.3 | 10 | 6.5 | 8 | 8.6 |
| **Localization** | nasopharynx | 4 | 1.6 | 3 | 2.0 | 1 | 1.1 |
|  | oropharynx | 79 | 32.1 | 56 | 36.6 | 23 | 24.7 |
|  | hypopharynx | 29 | 11.8 | 20 | 13.1 | 9 | 9.7 |
|  | oral cavity | 57 | 23.2 | 32 | 20.9 | 25 | 26.9 |
|  | larynx | 41 | 16.7 | 21 | 13.7 | 20 | 21.5 |
|  | parotid glands | 6 | 2.4 | 1 | 0.7 | 5 | 5.4 |
|  | other salivary glands | 3 | 1.2 | 3 | 2.0 | 0 | 0.0 |
|  | multi-level | 15 | 6.1 | 12 | 7.8 | 3 | 3.2 |
|  | others | 12 | 4.9 | 5 | 3.3 | 7 | 7.5 |
| **T-stage** | T1 | 35 | 14.2 | 22 | 14.4 | 13 | 14.0 |
|  | T2 | 53 | 21.5 | 33 | 21.6 | 20 | 21.5 |
|  | T3 | 64 | 26.0 | 44 | 28.8 | 20 | 21.5 |
|  | T4 | 86 | 35.0 | 49 | 32.0 | 37 | 39.8 |
| **N-stage** | N0 | 82 | 33.3 | 46 | 30.1 | 36 | 38.7 |
|  | N1 | 33 | 13.4 | 24 | 15.7 | 9 | 9.7 |
|  | N2 | 120 | 48.8 | 75 | 49.0 | 45 | 48.4 |
|  | N3 | 11 | 4.5 | 8 | 5.2 | 3 | 3.2 |
| **M-stage** | M0 | 232 | 94.3 | 143 | 93.5 | 89 | 95.7 |
|  | M1 | 10 | 4.1 | 8 | 5.2 | 2 | 2.2 |
| **UICC** | I | 24 | 9.8 | 14 | 9.2 | 10 | 10.8 |
|  | II | 20 | 8.1 | 13 | 8.5 | 7 | 7.5 |
|  | III | 48 | 19.5 | 32 | 20.9 | 16 | 17.2 |
|  | IVA/B | 145 | 58.9 | 87 | 56.9 | 58 | 6.2 |
|  | IVC | 9 | 3.7 | 7 | 4.6 | 2 | 2.2 |
| **Grading** | G1 | 6 | 2.4 | 3 | 2.0 | 3 | 3.2 |
|  | G2 | 157 | 63.8 | 96 | 62.7 | 61 | 65.6 |
|  | G3 | 74 | 30.1 | 49 | 32.0 | 25 | 26.9 |
|  | G4 | 1 | 0.4 | 1 | 0.7 | 0 | 0.0 |
| **HPV** | HPV-negative | 49 | 19.9 | 35 | 22.9 | 14 | 15.1 |
|  | HPV-positive | 34 | 13.8 | 23 | 15.0 | 11 | 11.8 |
|  | missing | 163 | 66.3 | 95 | 62.1 | 68 | 73.1 |
