# Supplementary material for: Radiotherapy for geriatric head-and-neck cancer patients: what is the value of standard treatment in the elderly?
Source: Radiat Oncol. 2020 Feb 4;15:31. doi: 10.1186/s13014-020-1481-z (PMC7001207; doi:10.1186/s13014-020-1481-z)
Supplement: Supplementary file 5 — Additional file 5: Table S2. Toxicity results after (chemo)radiotherapy of elderly patients with HNSCC according to the Common Terminology Criteria for Adverse Events (CTCAE) v5.0. [file 13014_2020_1481_MOESM5_ESM.docx]

| **Acute** | **n** | **%** |
| --- | --- | --- |
| CTCAE ≤ 2 | 108 | 43.9 |
| CTCAE 3 – 4 | 138 | 56.1 |
| CTCAE 5 | 0 | 0 |
| **Chronic** |  |  |
| CTCAE 0 | 31 | 13.7 |
| CTCAE 1 – 2 | 150 | 66.4 |
| CTCAE 3 – 4 | 45 | 19.9 |
| CTCAE 5 | 0 | 0 |
