# Supplementary material for: Radiotherapy for geriatric head-and-neck cancer patients: what is the value of standard treatment in the elderly?
Source: Radiat Oncol. 2020 Feb 4;15:31. doi: 10.1186/s13014-020-1481-z (PMC7001207; doi:10.1186/s13014-020-1481-z)
Supplement: Supplementary file 6 — Additional file 6: Table S3. Toxicity results consisting various (chemo)radiotherapy-related adverse reactions according to the Common Terminology Criteria for Adverse Events (CTCAE) v5.0. [file 13014_2020_1481_MOESM6_ESM.docx]

|  | **CTCAE grade** | | | | |  |
| --- | --- | --- | --- | --- | --- | --- |
| **Acute** | **0** | **1** | **2** | **3** | **4** | **5** |
| dermatitis | 76 | 63 | 83 | 19 | 0 | 0 |
| dysphagia | 52 | 36 | 75 | 80 | 0 | 0 |
| nausea | 182 | 14 | 23 | 22 | 0 | 0 |
| mucositis | 66 | 32 | 98 | 46 | 0 | 0 |
| xerostomia | 127 | 42 | 69 | 4 | 0 | 0 |
| hoarseness | 202 | 19 | 18 | 3 | 0 | 0 |
| dyspnea | 217 | 12 | 9 | 4 | 0 | 0 |
| dysgeusia | 117 | 29 | 96 | 0 | 0 | 0 |
| pain | 108 | 35 | 71 | 28 | 0 | 0 |
| cytopenia | 44 | 51 | 82 | 67 | 2 | 0 |
| weight loss | 41 | 57 | 37 | 9 | 0 | 0 |
| acute kidney injury | 236 | 0 | 0 | 10 | 0 | 0 |
| **Chronic** | **0** | **1** | **2** | **3** | **4** | **5** |
| skin toxicity | 184 | 28 | 7 | 0 | 0 | 0 |
| dysphagia | 107 | 27 | 54 | 31 | 0 | 0 |
| nausea | 201 | 4 | 4 | 2 | 0 | 0 |
| mucositis | 186 | 17 | 12 | 3 | 0 | 0 |
| xerostomia | 72 | 62 | 81 | 3 | 0 | 0 |
| hoarseness | 193 | 19 | 6 | 0 | 0 | 0 |
| dyspnea | 205 | 7 | 4 | 2 | 0 | 0 |
| dysgeusia | 102 | 54 | 62 | 0 | 0 | 0 |
| pain | 170 | 18 | 26 | 4 | 0 | 0 |
| cytopenia | 205 | 5 | 5 | 1 | 0 | 0 |
| renal insufficiency | 204 | 5 | 5 | 4 | 0 | 0 |
| jaw and dental injuries | 181 | 6 | 20 | 10 | 0 | 0 |
